# A Topological Model of the Hippocampal Cell Assembly Network

**DOI:** 10.3389/fncom.2016.00050

**Published:** 2016-06-02

**Authors:** Andrey Babichev, Daoyun Ji, Facundo Mémoli, Yuri A. Dabaghian

**Affiliations:** ^1^Jan and Dan Duncan Neurological Research Institute, Baylor College of MedicineHouston, TX, USA; ^2^Department of Computational and Applied Mathematics, Rice UniversityHouston, TX, USA; ^3^Department of Neuroscience, Baylor College of MedicineHouston, TX, USA; ^4^Department of Mathematics, Ohio State UniversityColumbus, OH, USA

**Keywords:** place cells, hippocampus, cell assemblies, cognitive map, topology

## Abstract

It is widely accepted that the hippocampal place cells' spiking activity produces a cognitive map of space. However, many details of this representation's physiological mechanism remain unknown. For example, it is believed that the place cells exhibiting frequent coactivity form functionally interconnected groups—place cell assemblies—that drive readout neurons in the downstream networks. However, the sheer number of coactive combinations is extremely large, which implies that only a small fraction of them actually gives rise to cell assemblies. The physiological processes responsible for selecting the winning combinations are highly complex and are usually modeled via detailed synaptic and structural plasticity mechanisms. Here we propose an alternative approach that allows modeling the cell assembly network directly, based on a small number of phenomenological selection rules. We then demonstrate that the selected population of place cell assemblies correctly encodes the topology of the environment in biologically plausible time, and may serve as a schematic model of the hippocampal network.

## 1. Introduction

**Figure 1:**
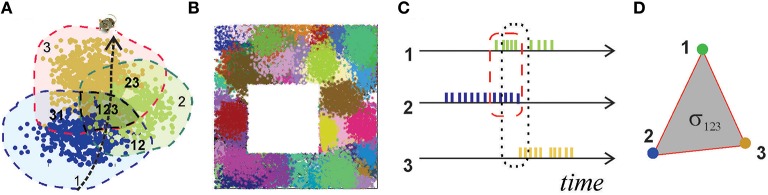
**Place fields and place cells**. **(A)** The blue, green, and brown dots, corresponding to the spikes produced by three different place cells, form well-defined spatial clusters, which represent their respective place fields. Spikes are positioned in space according to the animal's coordinates at the time of spiking. **(B)** A place field map produced by an ensemble of 300 place cells with mean peak firing rate *f* = 20 Hz and mean place field size *s* = 14 cm located in a 1 × 1 m environment. **(C)** A short time segment of the spike trains produced by three place cells. The periods of the cells' coactivity, marked by dashed lines, indicate overlap of their respective place fields **(A)**: cells *c*_1_ and *c*_2_ are coactive in the region 12, cells *c*_1_, *c*_2_, and *c*_3_ are co-active in the region 123. **(D)** A simplex σ_123_ represents schematically the spatial connectivity encoded by the coactivity of cells *c*_1_, *c*_2_, and *c*_3_. Its 1*D* edges correspond to pairwise coactivity, e.g., σ_12_ represents the coactivity of cells *c*_1_ and *c*_2_.

The mammalian hippocampus plays a major role in spatial learning by encoding a cognitive map of space—a key component of animals' spatial memory and spatial awareness (OKeefe and Nadel, 1978; Best et al., 2001). A remarkable property of the hippocampal neurons—the place cells—is that they become active only in discrete spatial regions—their respective place fields (Best and White, 1998) (Figure 1A). A number of studies have demonstrated that place cell activity can represent the animal's current location (Brown et al., 1998; Zhang et al., 1998), its past navigational experience (Carr et al., 2011; Derdikman and Moser, 2010), and even its future planned routes (Dragoi and Tonegawa, 2011; Pfeiffer and Foster, 2013). Numerical simulations suggest that a population of place cells can also encode a global spatial connectivity map of the entire environment (Curto and Itskov, 2008; Dabaghian et al., 2012; Arai et al., 2014). Hence, it is believed that the large-scale hippocampal representation of space emerges from integrating the information provided by the individual place cells, although the details of this process remain poorly understood.

Experimental studies point out that the hippocampal map is topological in nature, i.e., it is more similar to a subway map than to a topographical city map (Gothard et al., 1996; Leutgeb et al., 2005; Alvernhe et al., 2012; Dabaghian et al., 2014). This “topological” hypothesis has a major practical implication: it suggests that hippocampal data should be amenable to topological analyses, thereby allowing us to use powerful arsenals of computational topology. For example, the way place fields cover an environment calls to mind the Alexandrov-Čech theorem that holds that if a space *X* is covered with a sufficient number of discrete regions, then it is possible to reconstruct topology of *X* from the pattern of the overlaps between these regions. The argument is based on building a special simplicial complex N—the nerve of the cover—each *n*-dimensional simplex of which corresponds to a nonempty overlap of *n*+1 covering regions, and demonstrating that the topological signatures of N and *X* are same [for details see (Hatcher, 2002) and Methods in (Dabaghian et al., 2012)]. Since, the place cells' spiking activity induces a covering of the environment by the place fields, called a place field map [see Figure 1B and (Babichev et al., 2016)], the Alexandrov-Čech's theorem suggests that the place cells' coactivity (Figure 1C), which marks the overlaps of the place fields, may be used by the brain to represent the topology of the environment. The individual groups of coactive place cells, just like simplices, provide local information about the space, but together, as a neuronal ensemble, they represent space as whole—as the simplicial complex. This analogy establishes a possible approach to the long-sought connection between the cellular and system-scales, which was developed in (Dabaghian et al., 2012; Arai et al., 2014) into a working model of spatial memory. First, it was demonstrated that place cell coactivity can in fact be used to construct a *temporal* analog of the nerve complex, T, the simplexes of which, σ = [*c*_1_, *c*_2_, …, *c*_*k*_], correspond to the combinations of coactive place cells, *c*_1_, *c*_2_, …, *c*_*k*_ (Figure 1D). Second, using the methods of persistent homology (Zomorodian, 2005; Ghrist, 2008) it was shown that the topological structure of T captures the topological properties of the environment, if the range of place cell spiking rates and place field sizes happen to parallel biological values derived from animal experiments. Lastly, the persistent homology theory was used to estimate the rate of accumulation of global topological information, i.e., spatial learning.

However, it remained unclear whether it is possible to implement this algorithm in the (para)hippocampal network. On the one hand, electrophysiological studies suggest that place cells showing repetitive coactivity tend to form cell assemblies—functionally interconnected neuronal groups that synaptically drive a readout neuron in the downstream networks (Harris et al., 2003; Harris, 2005; Buzsaki, 2010; Huyck and Passmore, 2013)—which may be viewed as “physiological simplexes” implementing T. On the other hand, the place cell combinations of T are much too numerous to be implemented physiologically. In a small environment, c.a. 1 × 1 m, thousands of place cells are active and the activity of 50–300 of them is near maximal level at every given location (Buzsaki, 2010). The number of combinations of hundreds of coactive cells in an ensemble of thousands is unrealistically large, comparable to $C_{3000}^{100}\sim 10^{200}$. The number of cells in most parahippocampal regions, which may potentially serve as readout neurons, is similar to the number of place cells (Shepherd, 2004). This implies that only a small fraction of coactive place cell groups may be equipped with readout neurons, i.e., that the cell assemblies may encode only a small part of the place cell coactivities—those which represent a “critical mass” of spatial connections.

It is believed that place cells form as a result of competitive learning of inputs provided by the grid cells in the medial entorhinal cortex (MEC), which suggests a particular organization of functional connections between the grid cells and the place cells (Rolls et al., 2006; Solstad et al., 2006). Physiologically, the synaptic architecture of the place cell assembly network (which includes the readout networks downstream from the hippocampus) should also emerge from dynamically changing constellations of synaptic connections, which are commonly studied in terms of the synaptic and structural plasticity mechanisms (Chklovskii et al., 2004; Ghalib and Huyck, 2007; Wennekers and Palm, 2009; Itskov et al., 2011; Caroni et al., 2012). For a better understanding of the qualitative properties of the cell assembly network we propose a biologically plausible phenomenological approach that allows selecting the most prominent combinations of coactive place cells directly and demonstrate that the resulting population of cell assemblies is sufficient for representing the topology of the environment.

We proceed as follows: we start by outlining our methods and formulating general requirements to the model. Then we test three approaches to building a cell assembly network. First, we demonstrate that a “naïve” selection of the cell groups that show repetitive coactivity fails to produce a working cell assembly network. Then we propose two alternative methods of constructing the cell assembly network that reliably capture the topology of the environment. General implications of the approach and possible physiological connections are outlined in the Discussion.

## 2. The methods

**Figure 2:**
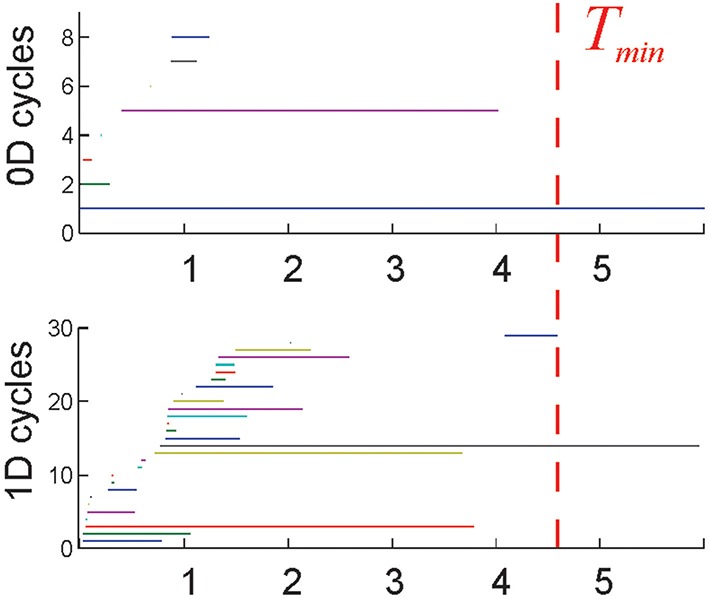
**Topological loops: each horizontal bar represents the timeline of a topological cycle in T(*T*): 0*D* loops (connectivity components) and the 1*D* loops**. Most cycles last over a short time before disappearing. A few remaining, *persistent* loops express stable topological information that may correspond to physical obstacles in the rat's environment. The time required for the correct number of cycles to appear is interpreted as the minimal time *T*_min_ required for the rat to learn the environment. The environment used in these simulations (Figure 1B) is topologically connected (*b*_0_ = 1), and has one central hole (*b*_1_ = 1), and no topological signatures in higher dimensions (*b*_*n*>1_ = 0). Thus, the topological barcode of this environment—the list of Betti numbers (*b*_0_, *b*_1_, *b*_2_,…)—is (1,1, 0, …). The last spurious loop (blue 1*D* timeline on the bottom panel) disappears at about *T*_min_ = 4.6 min, which is the learning time in this case.

Mathematically, the task of identifying a subpopulation of coactive place cell combinations corresponds to selecting according to biologically motivated criteria a subcomplex T_0_ of the full *coactivity complex* T. The cell assemblies correspond to the *maximal* simplexes of T_0_, (i.e., the ones that are not subsimplexes of any other simplex), in contrast with the maximal simplexes of the coactivity complex, T, which can represent any largest combinations of coactive cells. The “cell assembly complex,” T_0_, should satisfy several general requirements:

- **Effectiveness**. In the readercentric approach (Buzsaki, 2010), each cell assembly drives a coincidence detector *readout neuron* in the downstream brain regions. Since the number of the readout neurons is comparable to the number of place cells, the total number of the maximal simplexes in T_0_, *N*_max_(T_0_), should be comparable to the number of its vertexes, *N*_*c*_(T_0_),
  $$N_{\text{max}}\left(T_{0}\right)\approx N_{c}\left(T_{0}\right).$$
  However, the algorithm for selecting T_0_ should reduce only the number of coactive place cell combinations and not the place cells themselves, meaning that the number of vertexes in T and in T_0_ should not differ significantly. In mathematical literature, the number of *k*-dimensional simplexes of a simplicial complex is usually denoted as *f*_*k*_, and the list *f* = (*f*_0_, *f*_1_, …, *f*_*d*_) is referred to as the complex's *f*-vector (Gromov, 1968). However, since in neuroscience literature the letter *f* is often used to denote firing rates, we denote the number of *k*-dimensional simplexes by *N*_*k*_. As a shorthand notation, we use *N*_max_ to denote the number of the maximal simplexes and *N*_*c*_ the number of 0-dimensional simplexes in a given complex.
- **Parsimony**. To avoid redundancy, only a few cell assemblies should be active at a given location. Conversely, the rat's movements should not go unnoticed by the hippocampal network, i.e., the periods during which all place cell assemblies are inactive should be short.
- **Contiguity**. A transition of the spiking activity from one cell assembly σ_*i*_ to another σ_*i*+1_ occurs when some cells in σ_*i*_ shut off and a new group of cells activates in σ_*i*+1_ (see Supplementary Movies). The larger is the subassembly σ_*i, i*+1_ = σ_*i*_∩σ_*i*+1_ that remains active during this transition (i.e., the more cells are shared by σ_*i*_ and σ_*i*+1_) the more contiguous is the representation of the rat's moves and hence of the space in which it moves. The overlap between a pair of consecutively active simplexes can be characterized by a contiguity index
  $$\xi =\frac{\dim\left(\sigma_{i}\cap \sigma_{i+1}\right)}{\sqrt{\dim\left(\sigma_{i}\right)\dim\left(\sigma_{i+1}\right)}},$$
  which assumes the maximal value ξ = 1 for coinciding cell assemblies and ξ = 0 for disjoint ones. In constructing a cell assembly complex, we expect that the mean contiguity over the simplexes in T_0_ should not be lower than in T.
- **Completeness**. The cell assembly complex T_0_ should capture the correct topological signatures of the environment, such as obstacles, holes, and boundaries. For example, the lowest dimensional 0*D* and 1*D* loops in T_0_ represent, respectively, the piecewise and the path connectivity of the environment, as they are captured by the place cell coactivity. This information should emerge from the “topological noise” in a biologically plausible time period, comparable to the time required to obtain this information via the full complex, T [see (Dabaghian et al., 2012; Arai et al., 2014) and Figure 2].

### 2.1. Place cell spiking

Place cell spiking is modeled as a time-dependent Poisson process with spatially localized rate

$$\lambda_{c}\left(r\right)=f_{c}e^{-\frac{{\left(r-r_{c}\right)}^{2}}{s_{c}^{2}}},$$

where *r* is a point in the environment, *f*_*c*_ is the maximal firing rate of a place cell *c*, and *s*_*c*_ defines the size of the corresponding place field centered at *r*_*c*_ (Barbieri et al., 2004). In a familiar environment, the place fields are stable, that is, the parameters *f*_*c*_, *s*_*c*_, and *r*_*c*_ remain constant (Wilson and McNaughton, 1993; Brown et al., 2001). In our simulations, all computations were performed for 10 place cell ensembles, each containing 300 neurons with an ensemble mean maximal firing rate of 20 Hz and a mean place field size of 30 cm. The place field centers in each ensemble were randomly scattered across the environment and most quantities reported in the Results were averaged over ten place field configurations.

### 2.2. Spatial map

**Figure 3:**
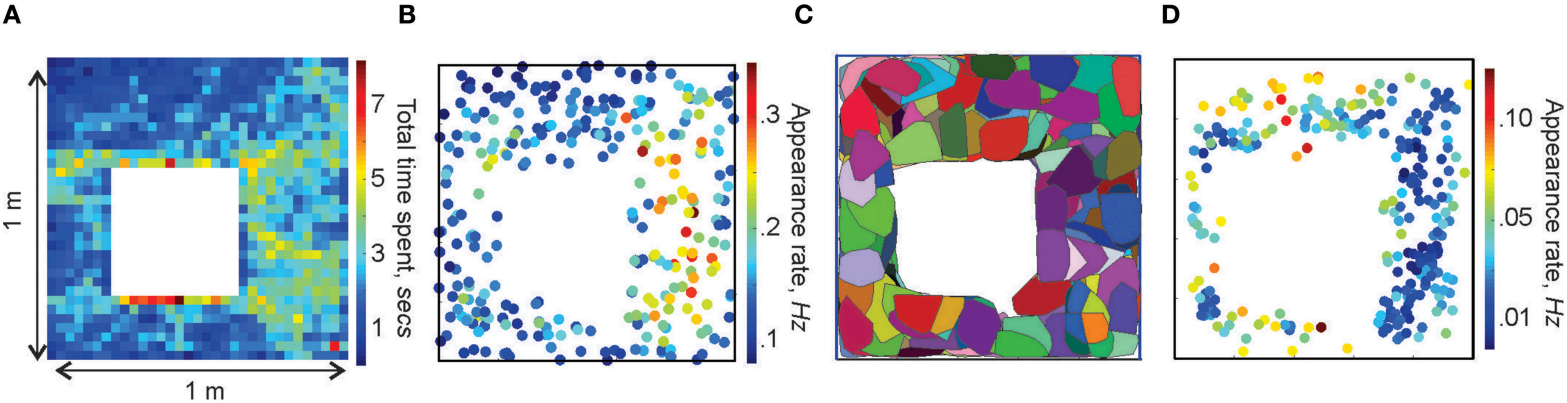
**Spatial maps**. **(A)** Occupancy of spatial locations in a 1 × 1 m environment—a 2*D* histogram of the time spent by the animal in different locations. **(B)** Frequency of place cells' spiking: each dot marks the location of a place cell's center *r*_*c*_ and indicates the corresponding appearance rate according to the colorbar. Higher appearance rates appear in the domain where the spatial occupancy is higher. **(C)** Simplex field map. The place field map for the same place cell ensemble is shown in Figure 1B. **(D)** Spatial distribution of the frequency of the maximal simplexes' appearances. Notice that, since place cells with higher appearance rates tend to produce higher order cell assemblies, which, in turn, have lower appearance rates, the spatial distribution of rates on **(B,D)** are complementary.

We simulated the rat's movements through a small (1 × 1 m) planar environment (Figure 1B), similar to the arenas used in typical electrophysiological experiments [see Methods in (Dabaghian et al., 2012)] over *T* = 25 min—the duration of a typical “running session.” The spatial occupancy rate of the rat's trajectory (i.e., the histogram of times spent at a particular location) and the frequency of the place cells' activity are shown on Figures 3A,B. The mean speed of the rat is 20 cm/sec, so that turning around the central obstacle takes about 7 s.

By analogy with the place fields, we designate the spatial domain where a combination of place cells comprising a simplex σ is active as its *simplex field*, *s*_σ_ (Figure 3C). If the simplex corresponds to a cell assembly, then *s*_σ_ may also be referred to as the *cell assembly field*. Similarly to the place fields and the place field map (Figure 1B), the collection of all simplex fields forms a *simplex field map* and the cell assembly fields form a *cell assembly map* (Figure 3C). These maps provide a better “geometric proxy” for the rat's cognitive map because they illustrate both the activity and the *co*activity of the individual place cells (Figures 3C,D). In the following, the structure of these maps will be used to discuss our selection algorithms. If the distinction between a cell assembly map and a simplex map is not essential, it will be referred to as a space map.

### 2.3. Population activity

**Figure 4:**
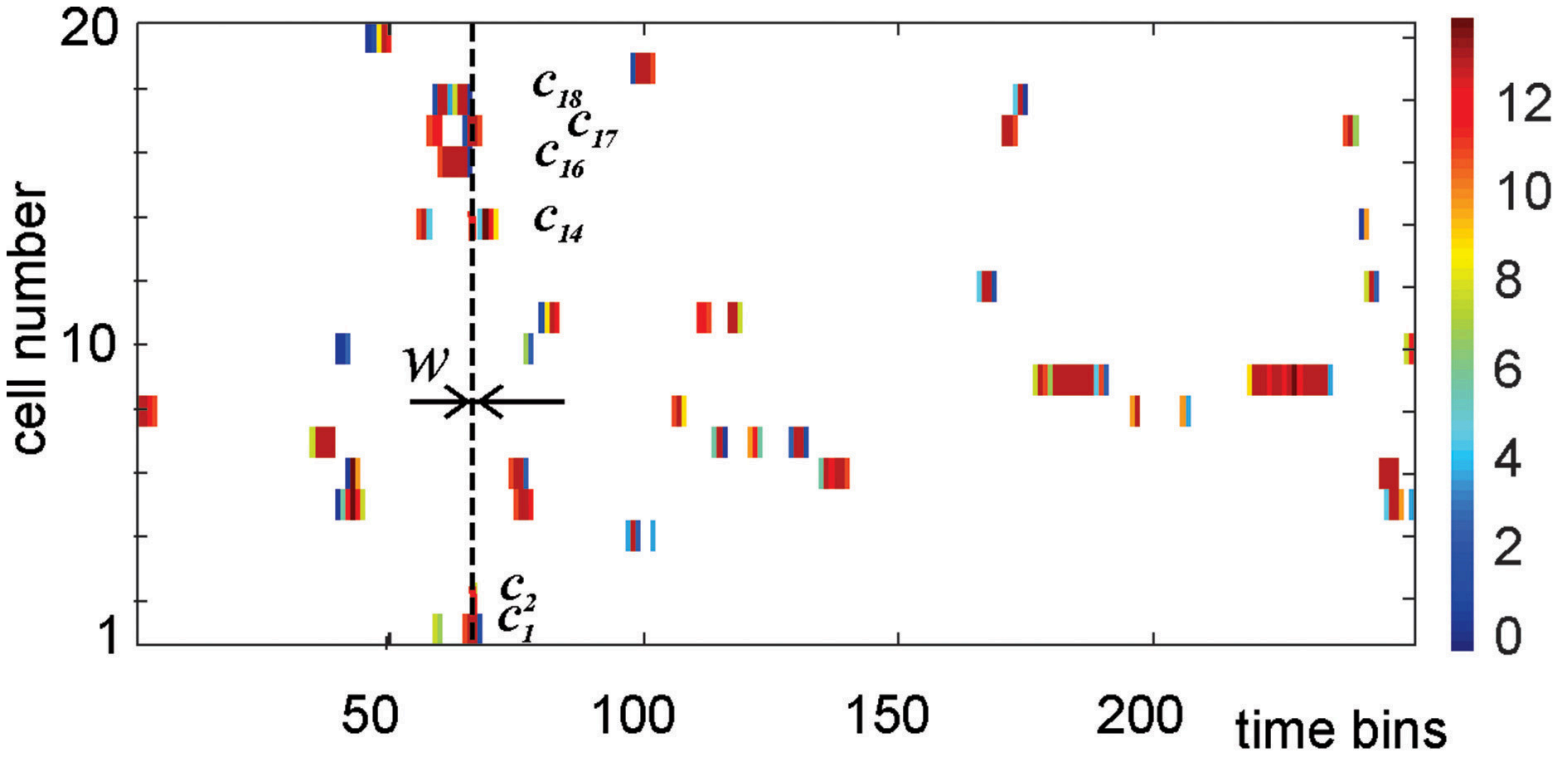
**An activity raster of a population of 20 place cells over 250 time bins**. Each row defines the activity vector of the corresponding place cell. The color of the ticks indicates the number of spikes contained in the corresponding bin of width *w*, according to the colorbar on the right. At every time step, the nonempty bins in the vertical column define the list of currently active cells, i.e., the active simplex σ_*t*_. During the time interval marked by the vertical dashed line, cell *c*_1_, *c*_2_, *c*_14_, *c*_16_, *c*_17_, and *c*_18_ are coactive, producing the coactivity simplex of fifth order σ = [*c*_1_, *c*_2_, *c*_14_, *c*_16_, *c*_17_, *c*_18_].

To define the population code (Pouget et al., 2000) of place cell combinations, we construct place cell *activity vectors* by binning spike trains into *w* = 1∕4 s long time bins [for a physiological justification of this value see (Mizuseki et al., 2009; Arai et al., 2014)]. If the time interval *T* splits into *n* such bins, then the activity vector of a cell *c* is

$$m_{c}\left(T\right)=\left[m_{c,1},\ldots ,m_{c,n}\right],$$

where *m*_*c, k*_ specifies how many spikes were fired by *c* in the *k*^*th*^ time bin. The components of *m*_*c*_, normalized by the total number of spikes, *M*_*c*_, define spiking probabilities, *p*_*c, k*_ = *m*_*k*_∕*M*_*c*_ (Perkel et al., 1967). A stack of activity vectors forms an *activity raster* illustrated on Figure 4.

Two cells, *c*_1_ and *c*_2_, are *coactive* over a certain time period *T*, if the dot product of their activity vectors does not vanish,

$$m_{c_{1}}\left(T\right)\cdot m_{c_{2}}\left(T\right)\neq 0.$$

The component-wise or Hadamard product of two activity vectors

$$m_{c_{1},c_{2}}=m_{c_{1}}⊙m_{c_{2}}=\left[m_{c_{1},1}m_{c_{2},1},m_{c_{1},2}m_{c_{2},2},\ldots ,m_{c_{1},n}m_{c_{2},n}\right]$$

defines the *coactivity vector* of cells *c*_1_ and *c*_2_, which can also be viewed as the activity vector of the corresponding 1*D* simplex σ_12_ = [*c*_1_, *c*_2_], *m*_σ_12__ ≡ *m*_*c*_1_, *c*_2__. Similarly, the Hadamard product of *k* vectors,

$$\begin{array}{l} m_{\sigma_{12\ldots k}}=m_{c_{1},c_{2},\ldots ,c_{k}}=m_{c_{1}}⊙m_{c_{2}}⊙\ldots ⊙m_{c_{k}}, \end{array}$$

defines the *activity vector of the simplex* σ_12…*k*_ = [*c*_1_, *c*_2_, …, *c*_*k*_].

For each activity vector, *m*_σ_, we also define its bit array mapping into a binary *appearance vector*, *a*_σ_, which indicates during which time-bins the corresponding simplex σ has made its appearance, i.e., *a*_σ, *i*_ = 1 iff *m*_σ, *i*_ > 0. The *appearance rate*, *f*_σ_(*T*), of a simplex σ over a time interval *T*, is defined as the *L*_1_ norm of its appearance vector, averaged over that time interval,

$$f_{\sigma }\left(T\right)=\left(1∕T\right)\Sigma_{i}a_{\sigma ,i}.$$

These appearance vectors and appearance rates allow distinguishing the intrinsic physiological characteristics of place cells' spiking, e.g., their maximal firing rate, from the frequency with which these cells activate due to the rat's movements through their respective place fields. While the maximal firing rate of a typical place cell is about 15 Hz (Best et al., 2001), the frequency of their activation is much lower.

## 3. Results

The simulated ensembles of 300 place cells in the environment shown on Figure 1B produced a coactivity complex T with about *N*_max_ = 1000 maximal simplexes. Despite the high dimensionality of these simplexes (up to *D* = 35, mean $\overset{-}{D}=17$), the characteristic dimensionality of a facet shared by two consecutively active simplexes, σ_*i*_ and σ_*i*+1_, is relatively low, so that the mean contiguity of T is ξ = 0.6. This implies that, geometrically, if the simplexes of T are viewed as multidimensional tetrahedrons, the selected complex, T_0_(θ), assumes a highly irregular shape (Supplementary Figure 1A).

**Figure 5:**
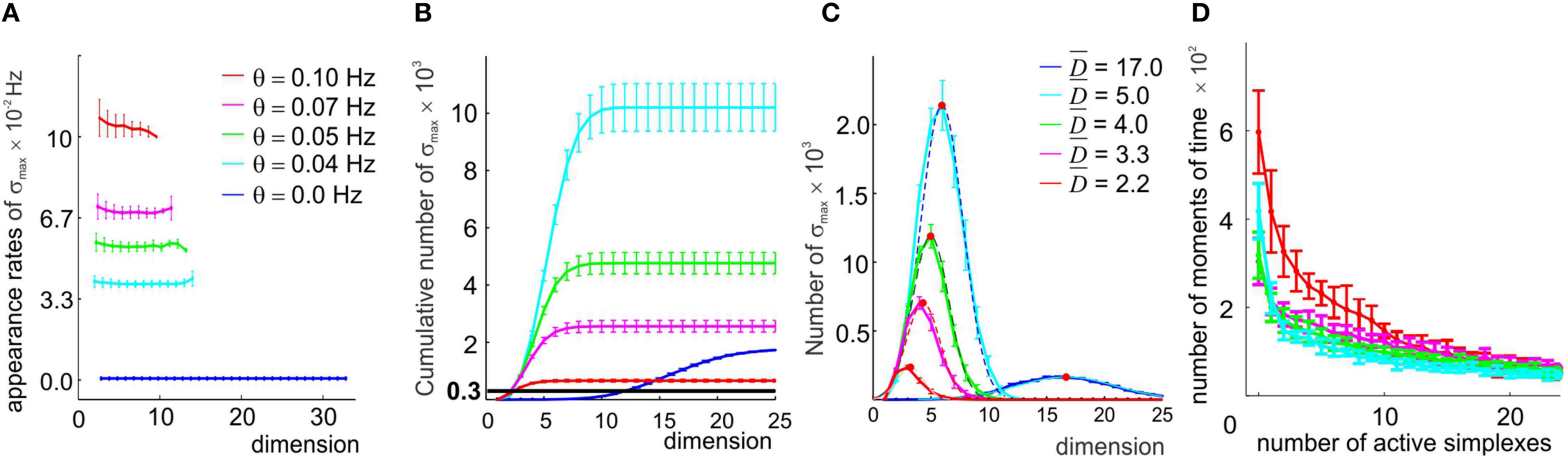
**A direct selection of the simplexes by appearance rates**. **(A)** In the original coactivity complex T(θ = 0), the maximal simplexes σ_max_ appear on average but once during the entire observation period, resulting in low appearance rates ($f_{\sigma }<10^{-3}$ Hz, blue line). Imposing four different thresholds θ (color coded) raises the appearance rates of the selected maximal simplexes almost uniformly in all dimensionalities. **(B)** Cumulative distribution of the number of maximal simplexes *N*_max_ over the selected simplexes' dimension. In the T(θ = 0) case *N*_max_ exceeds the number of vertexes (*N*_*c*_ = 300, black horizontal line) by almost an order of magnitude. Small threshold values result in an explosive increase of *N*_max_ which then begins to decrease for θ > 0.04 Hz, remaining significantly higher than *N*_*c*_ for all four tested values of θ. **(C)** The histograms of the maximal simplexes' dimensionalities fit with normal distribution. The high mean dimensionality ($\overset{-}{D}=17$) observed in the T(θ = 0) case reduces to $\overset{-}{D}=2.2$ for θ = 0.1 Hz. The width of the distributions is about 50% of $\overset{-}{D}$. **(D)** The histograms of the number of the coactive maximal simplexes, fit to an exponential distribution, demonstrate that the typical number of coactive simplexes is large, β > 10. All values are averaged over ten place field maps generated by 10 place cell ensembles with the same mean peak firing rate and mean place field size.

More importantly, nearly 100% of the maximal simplexes appeared only once during the entire 25 min period of navigation, i.e., a typical maximal simplex's appearance rate is low, $f_{\sigma }\sim 10^{-3}$ Hz. However, a typical vertex activated about 200 times or every seven seconds, suggesting that some of the lower dimensional subsimplexes may be better candidates for forming cell assemblies. Is it then possible to build a cell assembly complex T_0_ by discarding the high-dimensional maximal simplexes with low appearance rates and retaining their subsimplexes that appear more frequently? We tested this hypothesis by identifying the combinations σ whose coactivity exceeds a certain threshold *f*_σ_ > θ, and studied the properties of the resulting simplicial complex as a function of θ (Figure 5A).

First we observed that, as soon as the appearance threshold is introduced (θ≳10^−3^ Hz), the high dimensional simplexes start breaking up, releasing large numbers of lower dimensional subsimplexes: the number of *k*-dimensional subsimplexes in a *n*-dimensional simplex grows as combinatorial coefficient $C_{n+1}^{k+1}$, e.g., for *n* = 17 and *k* = 7, $C_{18}^{8}\approx 44,000$. As a result, the complex T_0_(θ) rapidly inflates. As θ increases further (θ > 0.04), the number of “passing” simplexes decreases, and T_0_(θ) begins to shrink in all dimensions (i.e., *N*_*D*_(θ_1_) > *N*_*D*_(θ_2_) for θ_1_ < θ_2_, for all *D*, (Figure 5B). Despite this, the number of maximal simplexes remains high: *N*_max_ = 30 × *N*_*c*_ at θ = 0.04 Hz, *N*_max_ = 7 × *N*_*c*_ at θ = 0.07 Hz and *N*_max_ = 3 × *N*_*c*_ for the highest tested threshold, θ = 0.1 Hz (Figure 5B), while their characteristic dimensionality drops from *D* = 17 to *D* = 7 at θ = 0.04 Hz and to *D* = 3 at θ = 0.1 Hz. The mean contiguity index for this range of thresholds remains close to ξ = 0.7, indicating that the degree of overlap between the selected combinations of place cells is higher than in the original coactivity complex.

However, raising the passing threshold θ quickly destroys the geometric integrity of the resulting complex's spatial map. As shown on Supplementary Figure 2, for θ = 0.05 Hz, only ~50% of the environment is covered by the remaining simplex fields, and for θ = 0.07 Hz the simplex map barely retains its one-piece connectedness: in some cases the complex T_0_ splits in two (the corresponding Betti numbers, *b*_0_, are listed in Supplementary Table 1, for an illustration see Supplementary Figure 3). For θ = 0.1 Hz, the complex fragments into multiple components (mean *b*_0_ ~ 7) that are riddled with holes: the Betti numbers *b*_*n*>0_ indicate the presence of hundreds of stable loops in higher dimensions. Thus, even if the coactive place cell combinations selected at θ ≥ 0.05 Hz could be supplied with readout neurons and would form cell assemblies, the resulting cell assembly network would not encode the correct spatial connectivity.

An additional problem is that reducing the order of the assemblies violates the “assembly code” for spatial locations: every time several subsimplexes σ_*i*_ are selected from a high-order maximal simplex σ, several overlapping simplex fields *s*_σ_*i*__ are produced in place of a single *s*_σ_. As a result, the parsimony of the representation is compromised: a location that was previously represented by a single simplex becomes represented by a few of its subsimplexes (Supplementary Figures 4A,B). Figure 5D shows a histogram of the numbers of simultaneously active maximal simplexes in T_0_: although most of the time only a few maximal simplexes are active, a coactivity of many of them (*n* > 25) is not uncommon. Conversely, while most of the time—on average 84% for the selected place cell ensembles—at least one simplex is active, longer inactivity periods are observed as described by double exponential distributed with the rate β ≈ 3.5 s (Supplementary Figure 2).

Overall, since most of the T_0_-requirements listed in the Methods fail, we are led to conclude that the most straightforward selection rule, based on selecting high appearance rates, does not produce the desired tradeoff between the order of the assemblies, the frequency of their appearances, and the quality of topological representation of the environment. This failure motivates the search for alternative methods.

### 3.1. Method I

To produce a more detailed approach to selecting coactive cell combinations, we observe that place fields are typically convex planar regions, and hence the existence of higher order overlaps between them actually follows from the lower order overlaps. According to Helly's theorem, a collection of *n* > *D*+1 convex *D*-dimensional regions in Euclidean space *R*^*D*^ will necessarily have a nonempty common intersection, if the intersection of every set of *D*+1 regions is nonempty (see (Eckhoff, 1993; Avis and Houle, 1995) and Supplementary Figure 4D). From the perspective of Čech theory, this implies that if *n* convex regions which cover a *D*-dimensional space contribute all the combinatorially possible *D*-dimensional simplexes to the nerve complex, then they also provide all the higher (up to *n* − 1) dimensional simplexes to it. In a planar (*D* = 2) environment, this implies that a set of four or more place fields has a common intersection, if any three of them overlap. Moreover, although mathematically it is possible that three place fields exhibit pairwise, but not triple overlap, the probability of such an occurrence is low (Supplementary Figure 4C). A direct computational verification shows that if a triple of place cells demonstrates pairwise coactivity, then, in over 90% of cases, it also correctly encodes a triple spatial overlap. In other words, a “clique” of pairwise coactivities indicates the overlaps of all higher orders, which implies that the spatial connectivity graph *G*_N_ whose vertexes correspond to the place fields and links represent pairwise overlaps, encodes most simplexes in the nerve complex N.

As a reminder, a clique of an undirected graph is a set of pairwise connected vertices. From the combinatorial perspective, a clique and a simplex have the same defining property: any subset of a simplex is its subsimplex and any subset of a clique is its subclique; a maximal clique is the one that is contained in no other clique. Hence, each graph defines its own “clique complex,” the *k*-dimensional simplexes of which corresponds to the graph's cliques with *k* + 1 vertices (Bandelt and Chepoi, 2008).

The observation that the nerve complex induced from the place field map can be approximated by the clique complex of the place field pairwise connectivity graph, suggests that the corresponding coactivity complex T can also be built based only on pairwise, rather than higher-order, coactivities. This approach is well justified physiologically, since pairwise coactivity detector pairs of synapses are commonly observed (Katz et al., 2007; Brette, 2012). The rule for defining the temporal analog of *G*_N_—the *relational graph* *G*_T_—is straightforward: a pair of vertexes is connected in *G*_T_ if the corresponding cells *c*_*i*_ and *c*_*j*_ are coactive. Thresholding pairwise coactivity rates according to the rule

(1) $$\begin{array}{l} C_{ij}=\{\begin{array}{ll} 1 & \text{if }f_{c_{i},c_{j}}\geq \theta \\ 0 & \text{if }f_{c_{i},c_{j}}<\theta . \end{array} \end{array}$$

allows constructing a family of relational graphs *G*_T(θ)_ over the pairs of place cells with high coactivity. The higher the threshold is, the sparser its connectivity matrix *C*_*ij*_ and the smaller the number of maximal cliques and hence of maximal simplexes in the corresponding clique complex. Since in the following the graph *G*_N_ will not be used we will suppress the subscript “T” in the notation for *G*_T_.

**Figure 6:**
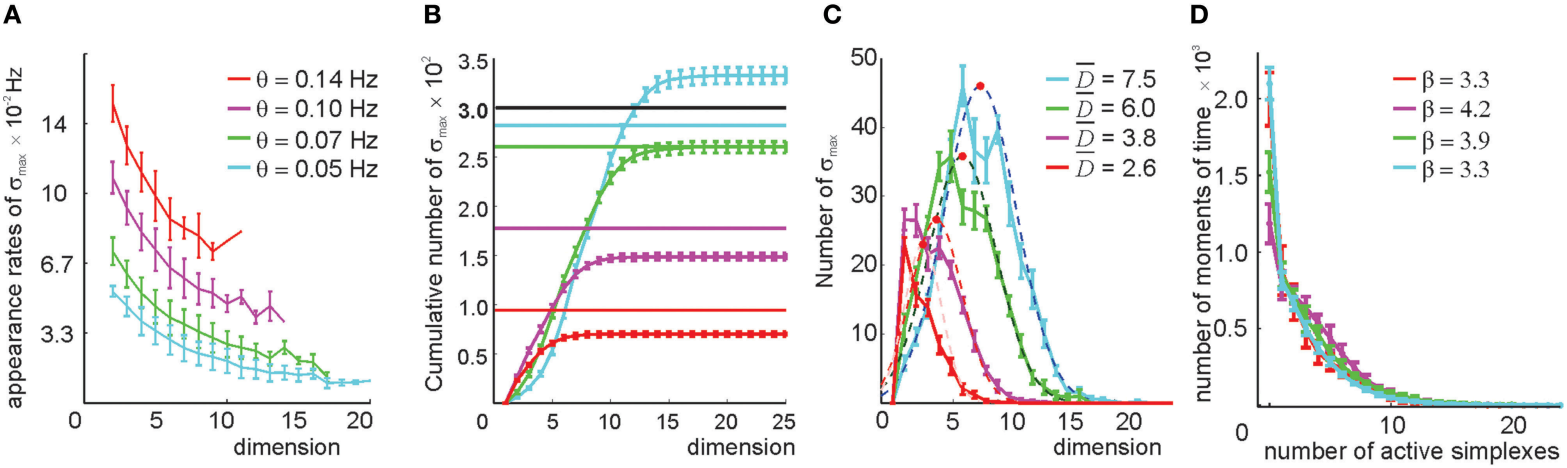
**Selecting maximal simplexes via the pairwise coactivity threshold (Method I)**. **(A)** The appearance rates of the maximal simplexes computed for four different pairwise appearance rate thresholds θ decrease as a function of their dimensionality. The values at *D* = 1 correspond to the value of the threshold imposed on the links' appearance rate. **(B)** Cumulative distribution of the numbers of maximal simplexes, *N*_max_, over the selected simplexes' dimension. The numbers of cells *N*_*c*_ for each threshold value are shown by horizontal lines. The tendency of the maximal simplexes to outnumber the vertexes *N*_max_ > *N*_*c*_, characteristic for small values of θ, is reversed around θ = 0.07 Hz, where *N*_max_ and *N*_*c*_ level out. **(C)** The histograms of the maximal simplexes' dimensionalities fit with normal distribution. The mean dimensionalities are similar to the ones produced by the previous selection method. The width of the distributions is about 50% of $\overset{-}{D}$. **(D)** The histogram of the number of coactive maximal simplexes, fit to an exponential distribution, shows that the expected number of coactive simplexes (β ~ 4) is significantly lower than in the previous selection method. The procedure of averaging over the place field maps is the same.

We studied the relational graphs *G*(θ) and their respective clique complexes T_0_(*G*(θ)) ≡ T_0_(θ) as a function of θ. First, we observed that the appearance rates of the maximal simplexes in T_0_(θ) become sensitive to the simplexes' dimensionality (Figure 6A), implying that this selection procedure in effect attributes different thresholds to simplexes of different dimensions by using only one free parameter θ. Second, the size of T_0_(θ) is not as large as before. As shown on Figures 6B–D, even for a relatively low threshold θ = 0.05 Hz, the number of maximal simplexes exceeds the number of cells only marginally. For higher thresholds, this number steadily decreases: *N*_*D*_(θ_1_) < *N*_*D*_(θ_2_) for θ_1_ > θ_2_ > 0.04 and *D* > 3, though in lower dimensions (1 ≤ *D* ≤ 3) this number may increase. The characteristic contiguity ranges between ξ = 0.65 at θ = 0.05 Hz to ξ = 0.72 at θ = 0.14 Hz, which is higher than the value produced by the direct simplex selection method. Geometrically, this implies that the collection of maximal simplexes selected by pairwise threshold selection is more aggregated than the collection produced via direct simplex selection, i.e., the resulting complex T_0_(θ) is geometrically more similar to a “simplicial quasimanifold” (see Supplementary Figure 1B). However, the number of place cells *N*_*c*_ drops as a result of discarding too many links with low appearance rate: *N*_*c*_ = 290 at θ = 0.05 Hz and *N*_*c*_ = 100 at θ = 0.14 Hz. At θ = 0.1 Hz number of cells levels out with the number of maximal simplexes, *N*_max_ ~ *N*_*c*_ = 260.

As before, raising the coactivity threshold degrades the spatial map. At θ > 0.07 Hz the simplex fields no longer cover the environment and at θ > 0.1 Hz the map fragments into pieces (Supplementary Figure 5). However, the resulting complex exhibits a much more regular topological behavior: the correct signature (*b*_0_ = 1, *b*_1_ = 1, *b*_2_ = 0, *b*_3_ = 0, …) in T_0_(θ) appears at θ = 0.05 Hz. The higher order Betti numbers (*b*_*n*≥2_) remain trivial at still higher θs (Supplementary Table 2A), even though the connectedness and path connectivity of the environment (*b*_1_ and *b*_0_) become misrepresented.

**Figure 7:**
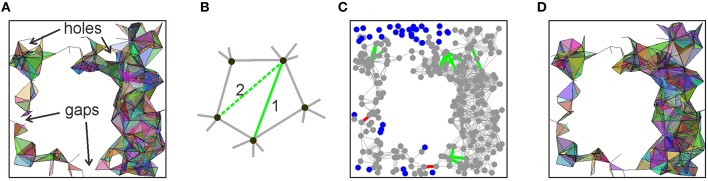
**Correction algorithms**. **(A)** A spatial projection of the 2*D* skeleton of T(θ) shows gaps and holes that compromise, respectively, the piecewise and path connectivity of T(θ). If the links across the gaps and holes of T(θ) are restored, then its correct connectivity structure may be regained. **(B)** A “hole” produced by five connected vertexes is closed by restoring some of the previously discarded crosslinks. **(C)** A projection of the relational graph *G* into the environment, shown in gray. The edges added across the gaps are shown in red and the edges added to fill the holes are shown in green. The vertexes that are left disconnected due to low appearance rates of the edges connecting them to other vertexes are shown by blue dots. **(D)** A spatial map of the resulting “patched” 2*D* skeleton of T(θ). The parameter values are *n*_*g*_ = 15, *m*_*h*_ = 10, and the lowered threshold for reintroducing the missing links is θ = 50.

This improvement of the behavior of T_0_(θ) suggests that, despite all the shortcomings, the link-selection strategy may lead to a successful model of the place cell assembly network. After all, it is not surprising that a single selection rule does not resolve all the aspects of the cell assembly formation. Yet if it captures the essence of the process, it should be possible to correct or to adjust its outcome. For example, one of the difficulties faced by the coactivity selection algorithm is that, for high θ, T_0_(θ) may brake into several pieces. However, the gaps between them are small. Thus, if a few discarded edges of the relational graph that originally bridged these gaps are retained, then the connectedness of T_0_(θ) may be spared (Figure 7A). Similarly, a “hole” in the relational graph is a linear chain of edges, connected tail to tail, with no shortcuts. However, if the links with the lower appearance rate (*f* ≥ θ_*h*_, θ_*h*_ < θ) that span across the hole exist at θ = 0, then they also can be restored (Figure 7B). This may remove the non-contractible chains of 1*D* simplexes in T_0_(θ) that compromised its path connectivity (Figures 7C,D). Thus, we implemented the following two *rectification algorithms*:

1. **Filling gaps**: find pairs of vertexes *v*_*a*_ and *v*_*b*_ separated in *G*(θ) by more than *n*_*g*_ edges and then test whether these vertexes are connected directly by links (from *G*(θ = 0)) whose appearance rate exceeds a lower threshold θ_*g*_ < θ. If such links exist, add them to *G*(θ) (red lines on Figure 7C).
2. **Closing holes**: A closed chain containing *m*_*h*_ ≥ 4 edges in *G*(θ), with no shortcuts, is likely to produce a hole in T_0_(θ). We identified such chains and restored the discarded cross-links whose appearance rate exceeds a lower threshold θ_*h*_ < θ.

Thus, both rectification algorithms depend on two parameters: the length of the involved chains (*n*_*g*_ for gaps and *m*_*h*_ for holes) and the value of the reduced threshold θ_*g*_ and θ_*h*_. In our numerical experiments, we found that the optimal value for the thresholds is θ_*h*_ = θ_*g*_ = 50, and the parameters range between 5 and 10 (*m*_*h*_) and 10 and 15 (*n*_*g*_). Typically, each rectification procedure is applied once or twice before the right signature of T_0_(θ) is achieved, and this without producing significant changes of the complex's structure, such as altering the appearance of its simplexes or increasing its size *N*_max_. As illustrated in the Supplementary Table 2B, the correct signature in the “repaired” complex is achieved for all cases at θ = 0.07 Hz. In particular, at θ = 0.07 Hz we obtain a simplicial complex T_0_ with the correct signature, having *N*_*c*_ = 260 vertexes and about the same number of maximal simplexes, *N*_max_ ≈ *N*_*c*_. These maximal simplexes appear on average at a rate of *f*_σ_ ≥ 0.07 Hz, at least during every other run of the rat around the environment, and have dimensionality *D* = 6. As a result, the requirements to T_0_ are met and the maximal simplexes of T_0_ may represent hippocampal place cell assemblies that together encode a map of the environment, and hence T_0_ itself can be viewed as the “cell assembly complex.”

### 3.2. Method II

A common feature of the appearance-rate-based selection rules is that the resulting simplicial complex reflects biases of its spatial occupancy: higher dimensional maximal simplexes concentrate over the parts of the environment where the rat appears more frequently. In particular, the relational graph shows a higher concentration of edges over the eastern segment of the environment (Figure 7C) where the occupancy rate is highest (Figure 3A). On the one hand, this is natural since the frequency of the place cells' spiking activity certainly does depend on the frequency of the rat's visits to their respective place fields, which therefore affects the hippocampal network's architecture (Chklovskii et al., 2004; Caroni et al., 2012). In fact, this argument is at the core of the classical “hippocampus as a cognitive graph” model (Burgess and O'Keefe, 1996; Muller et al., 1996), which proposes that the architecture of the hippocampal network is an epiphenomenon of the place cell coactivity. On the other hand, the physiological processes that produce synaptic connections may be more autonomous. For example, the CA3 region of the hippocampus is anatomically a recurrent network of place cells whose spiking activity and synaptic architecture are dominated by the network's attractor dynamics (Tsodyks, 2005; Wills et al., 2005; Colgin et al., 2010).

**Figure 8:**
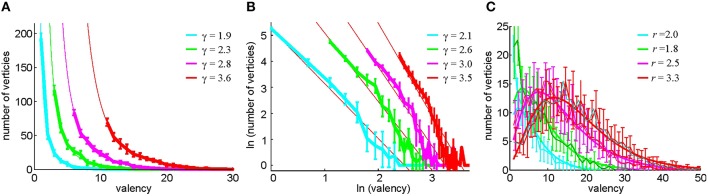
**Statistics of the vertex degrees in relational graphs**. **(A)** The histogram of the vertex degrees *k* in the neighbor-controlled relational graph *G*(*n*_0_), computed for *n*_0_ = 2, 4, 7, 12 (Method II) and fitted to a power law distribution *P*(*k*) ~ *k*^−γ^. The graph demonstrates that *G*(*n*_0_) is a scale-free network. **(B)** The same distribution on the log-log scale and an independent linear fit of the powers γ. The confidence intervals of the two fits, ranging between ±0.15 and ±0.3, overlap for each case. **(C)** In the pairwise coactivity threshold (Method I), the histogram of the relational graph's vertex degrees is fit by negative binomial distribution, suggesting that *G*(θ) is similar to a random network.

These considerations lead us to test an alternative method of constructing the relational graph based on selecting, for every cell, its *n*_0_ closest neighbors as defined by the pairwise coactivity rate *f*_*c*_*i*_, *c*_*j*__. Note that the resulting number of connections may be different for different cells: a cell *c*_1_ may be among the *n*_0_ closest neighbors of a cell *c*_2_, and hence *c*_1_ and *c*_2_ become connected, but the set of *n*_0_ closest neighbors of a cell *c*_1_ may not include *c*_2_, which bears a certain resemblance to the preferential attachment models (Barabasi and Albert, 1999). As a result, the vertex degrees *k* of the (undirected) relational graph may differ from one another and from *n*_0_. A direct computational verification shows that *k* is distributed according to a power law, *P*(*k*) ~ *k*^−γ^, where γ ranges, for different *n*_0_, between γ ~ 2 and γ ~ 4 (Figure 8), which implies that *G*(*n*_0_) demonstrates scale-free properties (Barabasi and Albert, 1999; Albert and Barabasi, 2002) characteristic of the hippocampal network (Bonifazi et al., 2009; Li et al., 2010). In contrast, the histogram of the vertex degrees in the threshold-controlled relational graph *G*(θ) may be fit with the negative binomial distribution (Figure 8B), which indicates that *G*(θ) is similar to a random graph.

This neighbor-selection method for building the relational graph *G*(*n*_0_) has a number of other immediate advantages over the threshold-controlled construction of *G*(θ). For example, no cells are excluded from T_0_ due to the low appearance of the edges connecting to them. As a result, the simplex fields are distributed more uniformly (Supplementary Figure 6), which helps capture the correct piecewise connectedness of the environment.

**Figure 9:**
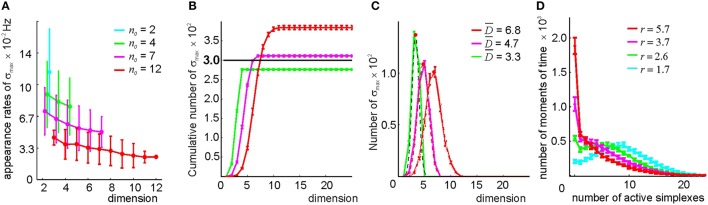
**Selecting maximal simplexes via best neighbor selection (Method II)**. **(A)** The appearance rates of the maximal simplexes in the simplicial complex T(*n*_0_), computed for four different values of *n*_0_ (color coded), decrease as a function of their dimensionality. **(B)** Cumulative distribution of the number of maximal simplexes *N*_max_ over the selected simplexes' dimension. For the tested values of *n*_0_, the fixed number of vertexes *N*_*c*_ = 300, indicated by the horizontal black line, is close to the number of maximal simplexes. For *n*_0_ = 7, the values *N*_max_ and *N*_*c*_ come closest. **(C)** The histograms of the maximal simplexes' dimensionalities, fit to the normal distribution, indicate that for the relational graph with a similar number of links, the mean dimensionalities of the maximal simplexes are smaller than in in the complex built via the threshold-selection method. The width of the distributions is about 40% of $\overset{-}{D}$. **(D)** The histogram of the number of coactive maximal simplexes, fit to a gamma distribution. An expected number of coactive simplexes ranges between *r* = 2 and *r* = 6. The procedure of averaging over the place field maps is the same.

By studying the properties of the clique complexes produced by the relational graphs *G*(*n*_0_) for *n*_0_ = 2, 4, 7, and 12—parameters chosen to produce similar numbers of edges as in the previous method—we found that the number of maximal cliques in T_0_(*G*(*n*_0_)) is typically lower than in T_0_(*G*(θ)). The appearance rates of maximal cliques in *G*(*n*_0_) are more scattered and less sensitive to dimensionality than in *G*(θ) (Figure 9A and Supplementary Figure 6). The number of maximal simplexes in T_0_(*n*_0_) remains close to the number of cells (Figures 9B,D) and their dimensionality is lower than in the threshold-based selection approach (Figure 9C). The contiguity index in all complexes ranges between to ξ = 0.67 and ξ = 0.71. The coverage of the space with the simplex fields improves with growing *n*_0_ (see Supplementary Figure 6)—for *n*_0_ > 2 the complex T_0_(*n*_0_) is connected, while the behavior of *b*_0_ is more regular (see Supplementary Table 3). However, the path connectivity of the complex T_0_(*n*_0_) remains deficient for all *n*_0_ because the number of stable spurious 1*D* loops remains high (Supplementary Table 3). After filling the gaps and closing the holes, most complexes constructed for *n*_0_ ≥ 7 acquire correct topological signatures (Supplementary Table 3), and the requirements to T_0_ are satisfied. Thus, the simplicial complex obtained by the neighbor selection method for *n*_0_ ≥ 7 can also be viewed as a “cell assembly complex,” meaning it can serve as a formal model of the place cell assembly network with mean contiguity ξ = 0.7.

## 4. Discussion

The hippocampal representations of the environment are based on the temporal organization of the place cells' spiking and on the mechanisms of processing the resulting spike trains in the downstream networks. While the place cells' spiking determinants [both spatial (O'Keefe and Burgess, 1996; Jeffery et al., 1997; Fenton et al., 2000) and nonspatial (Sharp et al., 1995; Wood et al., 2000)] are relatively well–studied, the readout mechanisms remain poorly understood. It is believed that groups of place cells exhibiting frequent coactivity form assemblies that jointly trigger spiking activity of their respective readout neurons, but the specific architecture of the cell assembly network has not been fully identified.

### 4.1. Descriptive vs. phenomenological approach

Previously, the topological approach was used to quantify the information encoded by the hippocampal network. Each individual group of coactive place cells, contributing *local* information about the space, was represented by a simplex, and hence the entire neuronal ensemble, encoding space as whole, was represented by a coactivity complex T (Curto and Itskov, 2008; Dabaghian et al., 2012; Arai et al., 2014). Specifically, the low order (pair and triple) coactivity events were used to construct the 2*D* skeleton of T, and then its 0*D* and 1*D* topological loops were matched with the topological loops in the environment. In the current work, the topological approach is extended to produce the functional architecture of the cell assembly network—schematically represented by a cell assembly complex T_0_—and relating its structure to the net topological information it encodes. In contrast with T, the maximal simplexes of T_0_ are viewed as representations of the physiological place cell assemblies, rather than any largest combinations of the coactive place cell groups. In particular, the model allows constructing the higher order assemblies that may potentially represent both the low-dimensional spatial environment and the high-dimensional memory space (Eichenbaum et al., 1999; Buzsaki, 2010). Importantly, the learning times *T*_min_ estimated from the dynamics of the 0*D* and 1*D* loops in T_0_ remain close to the learning times computed for the full coactivity complex T (see Supplementary Table 4). This implies that the selected, “core” pool of coactive place cell combinations captures the topological structure of the environment as fast and as reliably as the entire set of the place cell coactivities.

### 4.2. Physiological connections

In order to elicit an action potential, the impinging spikes must conjointly hyperpotentiate the readout neuron. A qualitative insight into this process was suggested by (Jarsky et al., 2005), who demonstrated that a pair of impinging synapses can “gate” one another: if a synaptic input *s*_2_ comes within a short period *w* after the synaptic input *s*_1_, then together these two inputs can polarize a large segment of the dendritic tree, which may lead to hyperpolarization of the entire postsynaptic neuron. This mechanism can be viewed as a physiological implementation of the “coincidence detection” for a pair of inputs and one can immediately see how it could be used to detect a larger (*k* > 2) number of inputs. One can think of the *k* nearly-simultaneous *individual* inputs *s*_*i*_ as of *k*(*k* − 1)∕2 nearly-simultaneous *pairs* of inputs (*s*_*i*_,*s*_*j*_), each one of which polarizes a particular fragment *d*_*ij*_ of the dendritic tree. If the physiology of the readout neuron is such that it hyperpotentiates only in response to nearly simultaneous inputs (*s*_*i*_,*s*_*j*_), then the readout neuron functions as a coincidence detector. In contrast, if the dendritic tree can retain the local polarizations over an extended period ϖ > *w*, then such neuron will integrate low order inputs over that time.

The links in the coactivity graph G can be viewed as schematic representations of the pairs of potentiating synapses: the proposed Methods I and II represent two possible ways of selecting the most “prominent” pairs. In Method I, the “winning pairs” of mutually gating synapses are selected based on the frequency of their appearance. Alternatively, given the number *n*_0_ of connections that a given cell can produce (based, e.g., on the number of axon terminals), one can aim to select these connections optimally—this is Method II.

### 4.3. Developments

We view the proposed algorithms as basic models of a general “phenomenological” approach that can be further developed along several broad lines. First, the structure of the relational graph is currently deduced from the activity vectors defined over the entire navigation period *T* = 25 min. A biologically more plausible selection algorithm should be adaptive: the structure of the relational graph at a given moment of time *t* < *T* should be based only on the spiking information produced before *t*. Hence, in a more advanced model, the structure of the relational graph should develop in time, and in general the cell assemblies comprising T_0_ should be derived using synaptic and structural plasticity mechanisms. Second, the selection criteria in Methods I and II above may be individualized: the appearance threshold used to construct the relational graph can be assembly-specific, i.e., θ = θ(σ), so that the properties of the resulting network would be described in terms of the probability distribution of the threshold values across the cell assembly population. Similarly, the number of neighbors can be made cell-specific, *n*_0_ = *n*_0_(*c*_*i*_), which should permit better control over the topological properties both of the network and of the cell assembly complex. Third, threshold control can be implemented using different coactivity metrics, for instance via the pairwise correlation coefficient

(2) $$\begin{array}{l} \rho \left(c_{1},c_{2}\right)=\left(m_{1}\cdot m_{2}\right)∕|m_{1}||m_{2}|, \end{array}$$

which would connect cells with correlated spiking (irrespective of their firing rates), in contrast with the metric (2), which does the opposite. In general, two metrics ρ and ρ′, produce relational graphs with different topologies. Nevertheless, they may produce similar or identical large-scale effects, such as generating topologically identical cell assembly complexes T_0_, or exhibit similar learning times, *T*_min_. Identifying classes of metrics that produce topologically similar results will be examined in future research.

## Author contributions

All authors listed, have made substantial, direct and intellectual contribution to the work, and approved it for publication.

## Funding

The work was supported in part by Houston Bioinformatics Endowment Fund, the W. M. Keck Foundation grant for pioneering research and by the NSF 1422438 grant.

### Conflict of interest statement

The authors declare that the research was conducted in the absence of any commercial or financial relationships that could be construed as a potential conflict of interest.
