# Supplementary material for: A Topological Model of the Hippocampal Cell Assembly Network
Source: Front Comput Neurosci. 2016 Jun 2;10:50. doi: 10.3389/fncom.2016.00050 (PMC4889593; doi:10.3389/fncom.2016.00050)
Supplement: Supplementary file 1 [file Presentation1.pdf]

Supplementary Material: A Topological Model of the Hippocampal Cell Assembly Network

Andrey Babichev, Daoyun Ji, Facundo Mémoli and Yuri Dabaghian*
*Correspondence:
Yuri Dabaghian
dabaghia@bcm.edu

1 SUPPLEMENTARY FIGURES

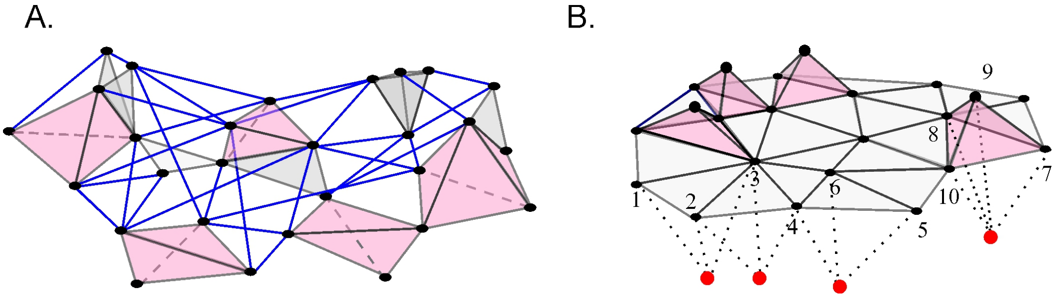

Supplementary Figure 1. Simplicial complexes. (**A**) A schematic representation of an irregular simplicial complex $\mathcal{K}$, in which the number of maximal simplexes is larger than the number of vertexes (black dots). The maximal $1D$ simplexes are shown as blue segments, the $2D$ simplexes as gray triangles and the $3D$ simplexes as pink tetrahedrons. (**B**) A simplicial "quasi-manifold," $\mathcal{Q}$, which has a similar number of vertexes and maximal simplexes of different dimensionalities. If each maximal simplex, e.g. $(1, 2, 3)$ or $(10, 7, 8, 9)$, corresponds to an assembly of place cells driving a readout neuron (red dots), then $\mathcal{Q}$ is a cell assembly complex. Dotted lines represent synaptic connections from the place cells to the readout neuron.

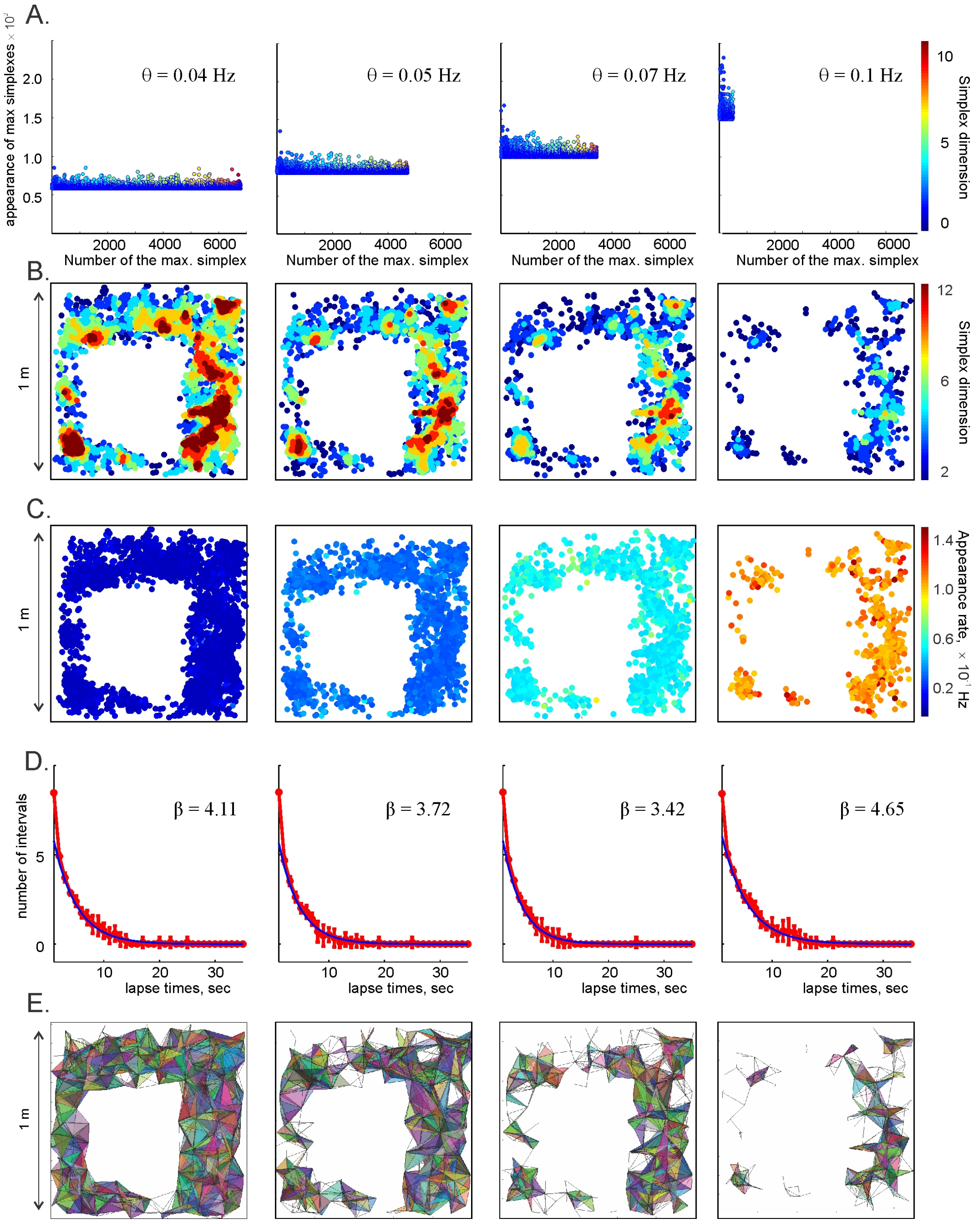

Supplementary Figure 2. The simplicial complexes $\mathcal{T}_0(\theta)$ constructed by direct selection of coactive combinations, for four different values of θ. (**A**) The appearance rates of simplexes, arranged from left to right according to their dimension. Each dot corresponds to a maximal simplex whose dimension is color-coded according to the colorbar on the right. (**B**) Spatial distribution of the dimensionalities of the selected simplexes. (**C**) Spatial distribution of the appearance rates of the selected simplexes. (**D**) The histograms of the lapse times, fit to double exponential distribution (blue line), and the value of the fitted distribution's rate β. (**E**) Spatial projections of the $2D$ skeletons of the $\mathcal{T}_0(\theta)$. Data for all panels is computed for a specific place field map for illustrative purposes.

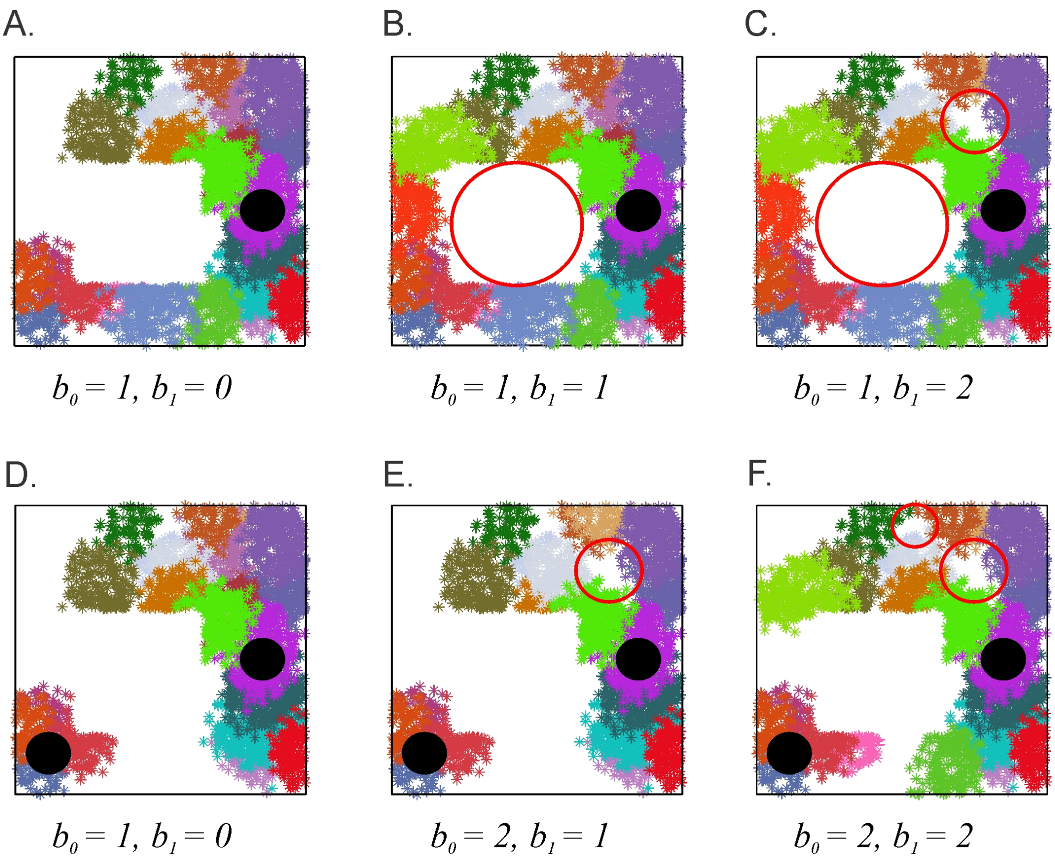

Supplementary Figure 3. Low-dimensional topological features of the cell assembly complexes captured by place field map. (**A**) A place filed map corresponding to a singly-connected ($b_0 = 1$, marked by the dot) complex with no non-contractible loops ($b_1 = 0$). (**B**) A place field map corresponding to a complex with correct list of Betti numbers (correct topological barcode (Ghrist, 2008)): the physical hole (see Figures 1 and 3) produces one non-contractible loop (red circle, $b_1 = 1$). (**C**) A map containing a spurious hole in the upper-right corner produces an extra persistent loop (the additional small circle, net $b_1 = 2$). (**D**) A map containing two disconnected pieces marked by the black dots (net $b_0 = 2$) and having no non-contractible loops ($b_1 = 0$). (**E**) A map containing two pieces and one persistent spurious $1D$ loop. (**F**) The green and the brown place fields at the top connect, yielding another persistent spurious $1D$ loop. Compare these illustrations and the topological barcodes to the illustrations and Suppl. Movies provided in (Singh et al. 2008)

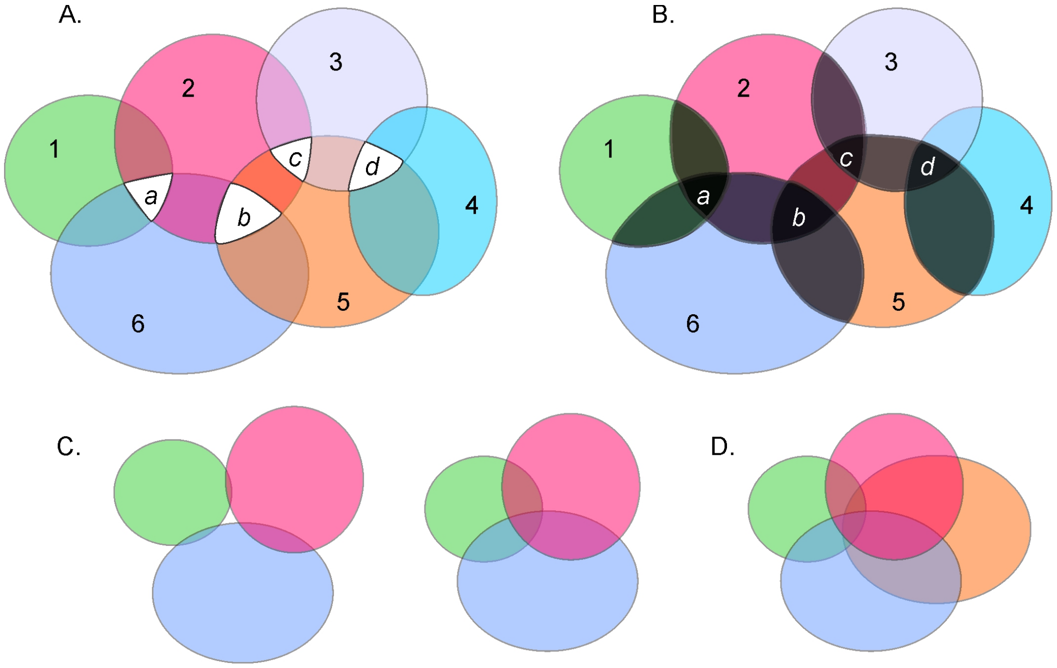

Supplementary Figure 4. Schematic illustrations of spatial maps. (**A**) Schematic representations of 3^d order simplex fields, a, b, c and d, encoded by third order maximal simplexes, $\sigma_a = (v_1, v_2, v_6)$, $\sigma_c = (v_2, v_3, v_5)$, etc. (**B**) If the $2D$ simplexes are discarded and their $1D$ faces are retained, then the second-order simplex fields are produced, shown here as overlapping shaded regions. The original simplex fields a, b, c and d are now represented by the coactivity of three pairs, e.g., a is represented by $\sigma_{a,1} = (v_1, v_2)$, $\sigma_{a,2} = (v_2, v_6)$ and $\sigma_{a,3} = (v_1, v_6)$. (**C**). The three place fields on the left exhibit pairwise, but not triple overlap. In the generic spatial configuration shown on the right, pairwise overlapping place fields also produce a triple overlap. (**D**) Four pairwise overlapping convex regions in $2D$ produce all the higher order (triple and quadruple) overlaps.

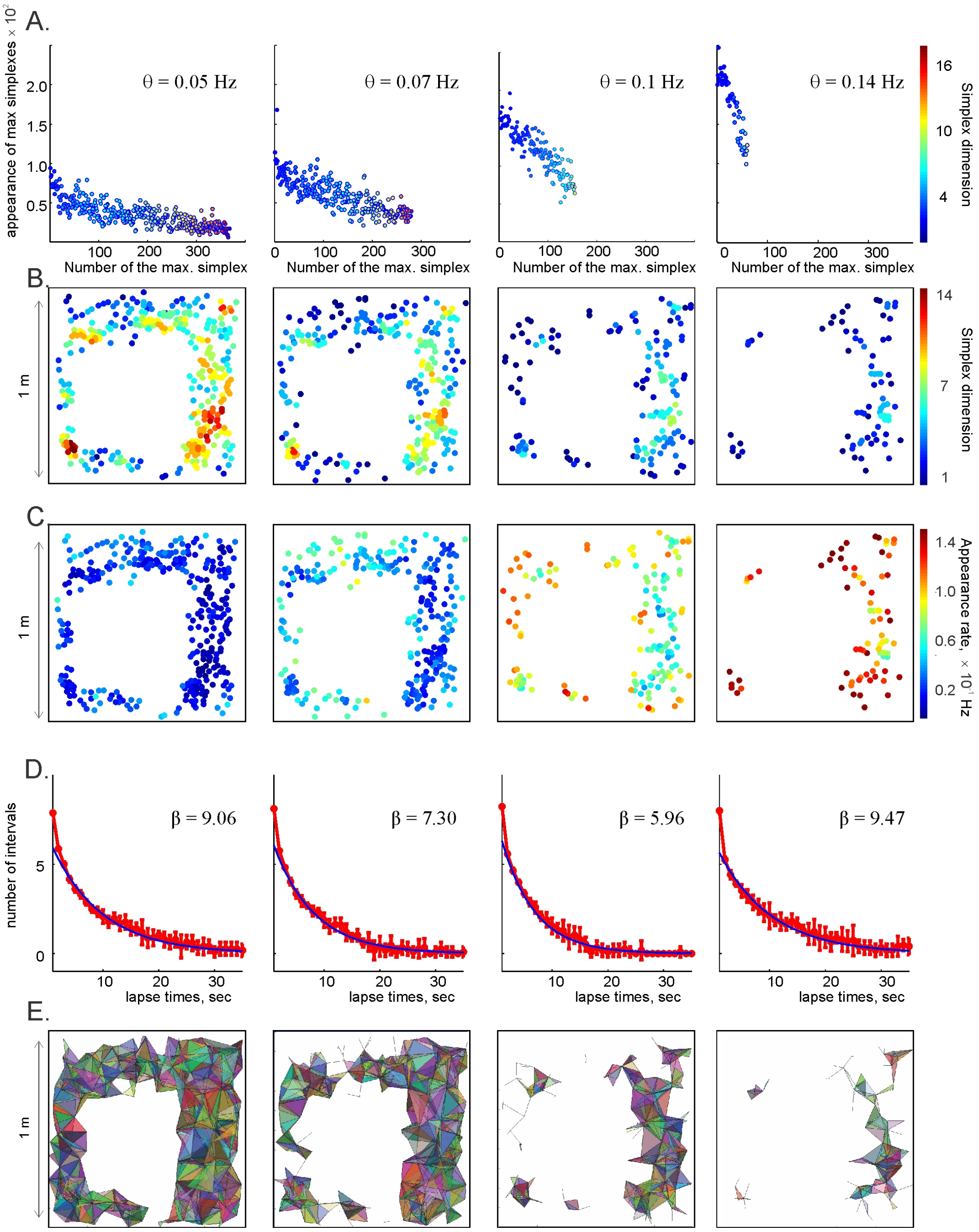

Supplementary Figure 5. Simplicial complexes $\mathcal{T}_0(\theta)$ constructed via pairwise coactivity selection (Method I) for four different threshold values. (**A**) The appearance rates of the maximal simplexes, arranged according to their dimension, demonstrate remarkably tight, graph-like distribution. The color of the dots corresponds to the dimension of the simplexes, as indicated by the colorbar on the right. (**B**) Spatial distribution of the dimensionalities of the selected simplexes. (**C**) Spatial distribution of the appearance rates of the selected simplexes. (**D**) The histograms of the lapse times, fit to double exponential distribution, and the value of the fitted distribution's parameter β. (**E**) Spatial projections of the $2D$ skeletons of $\mathcal{T}_0(\theta)$. Data for all panels is computed for a specific place field map for illustrative purposes.

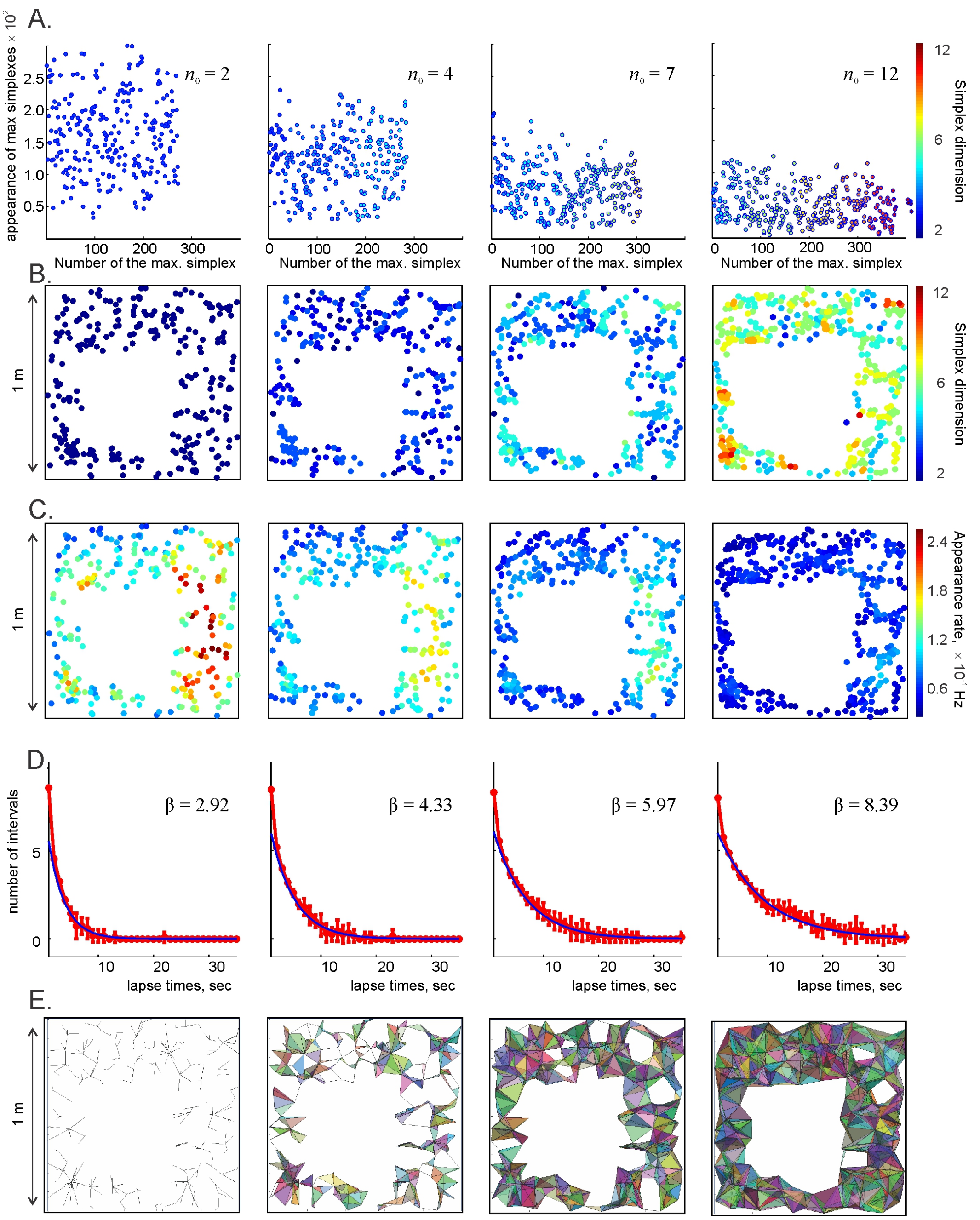

Supplementary Figure 6. The selected simplicial complexes $\mathcal{T}_0(n_0)$ constructed via the closed-neighbor selection algorithm (Method II) for four different values of n_0. (**A**) The appearance rates of simplexes, arranged according to their dimensions, color-coded as indicated by the colorbar on the right. (**B**) Spatial distribution of the dimensionalities of the selected simplexes. (**C**) Spatial distribution of the appearance rates of the selected simplexes. (**D**) The histograms of the lapse times, fit to double exponential distribution, and the value of the fitted distribution's parameter β. (**E**) Spatial projections of the $2D$ skeletons of $\mathcal{T}_0(n_0)$.

2 SUPPLEMENTARY TABLES

Supplementary Table 1. Topological signature of the complex selected by simplex rate thresholding. Each cell contains a list of four Betti numbers, (b_0, b_1, b_2, b_3). For the low rate, $f_\sigma = 0.04$ Hz, $\mathcal{T}_0(\theta)$ encodes the correct spatial connectedness of the environment ($b_0 = 1$). For intermediate rates, $0.05 \leq f_\sigma \leq 0.07$ Hz, $\mathcal{T}_0(\theta)$ may occasionally break into two pieces ($b_0 = 2$) and for $f_\sigma \geq 0.10$ Hz and higher, $\mathcal{T}_0(\theta)$ fragments into multiple components. In higher dimensions $D \geq 1$, $\mathcal{T}_0(\theta)$ contains over a hundred noncontractible topological loops.

θ = 0.04 Hz	θ = 0.05 Hz	θ = 0.07 Hz	θ = 0.1 Hz
1 139 509 569	1 171 393 410	2 198 324 214	5 131 108 15
1 126 409 480	1 175 353 286	1 203 247 144	7 147 71 17
1 156 580 853	2 211 518 571	2 233 330 419	6 139 152 41
1 135 407 551	1 176 369 333	1 201 296 152	7 139 50 7
1 149 422 585	2 172 342 453	2 185 312 320	13 122 107 73
1 130 485 775	1 186 447 392	1 190 335 250	4 164 84 27
1 106 459 655	1 202 352 603	1 177 395 353	8 179 117 25
1 120 519 787	1 194 397 449	1 215 333 237	8 160 109 63
1 131 441 757	1 165 459 499	1 203 339 197	8 149 65 15
1 160 364 575	1 226 313 368	1 175 270 222	8 117 92 14

Supplementary Table 2. Topological signature of the complex selected by link-rate thresholding. (**A**) For the low rate $f_\sigma = 0.05$ Hz, $\mathcal{T}_0(\theta)$ occasionally produces the correct topological signature ($b_0 = b_1 = 1$, $b_{n>1} = 0$, shown in bold). For intermediate rates $f_\sigma \sim 0.07$ Hz, $\mathcal{T}_0(\theta)$ may occasionally break into two pieces ($b_0 = 2$) and produce extra noncontractible loops in $1D$. For high thresholds, $f_\sigma \geq 0.10$ Hz, $\mathcal{T}_0(\theta)$ fragments into multiple components. However, the connectivity of $\mathcal{T}_0(\theta)$ in higher dimensions, $D \geq 2$, is correct for all cases, which implies that $\mathcal{T}_0(\theta)$ contracts into $2D$. (**B**) After applying the correction algorithms, the selected complexes acquire correct topological signature for $f_\sigma \leq 0.07$ for all maps. The corresponding learning times $T_{\min}$ are listed in Suppl. Table 4.

A. Original				B. Corrected			
θ = 0.05	θ = 0.07	θ = 0.1	θ = 0.14	θ = 0.05	θ = 0.07	θ = 0.1	θ = 0.14
**1 1 0 0**	2 1 0 0	5 1 0 0	3 0 0 0	**1 1 0 0**	**1 1 0 0**	1 0 0 0	3 0 0 0
1 2 0 0	1 5 0 0	7 2 0 0	7 1 0 0	**1 1 0 0**	**1 1 0 0**	1 0 0 0	2 0 0 0
1 2 0 0	2 1 0 0	6 2 0 0	7 2 0 0	**1 1 0 0**	**1 1 0 0**	1 0 0 0	1 0 0 0
**1 1 0 0**	1 2 0 0	7 7 0 0	7 1 0 0	**1 1 0 0**	**1 1 0 0**	1 0 0 0	1 0 0 0
**1 1 0 0**	2 3 0 0	13 1 0 0	8 1 0 0	**1 1 0 0**	**1 1 0 0**	1 0 0 0	3 0 0 0
1 3 0 0	1 0 0 0	4 6 0 0	11 0 0 0	**1 1 0 0**	**1 1 0 0**	1 0 0 0	1 0 0 0
2 1 0 0	1 5 0 0	8 1 0 0	7 5 0 0	**1 1 0 0**	**1 1 0 0**	2 0 0 0	3 0 0 0
1 2 0 0	1 2 0 0	8 2 0 0	9 1 0 0	**1 1 0 0**	**1 1 0 0**	1 0 0 1	2 0 0 0
1 3 0 0	1 4 0 0	8 2 0 0	9 2 0 0	**1 1 0 0**	1 1 1 0	**1 1 0 0**	4 0 0 0
1 3 0 0	1 5 0 0	8 2 0 0	7 6 0 0	**1 1 0 0**	**1 1 0 0**	2 0 0 0	3 0 0 0

Supplementary Table 3. Topological signatures of the complexes selected by the neighbor-selection algorithm. (**A**) If but one pair of closest vertexes is selected $n_0 = 2$, $\mathcal{T}_0(n_0)$ breaks into multiple components. For $n_0 \geq 5$, $\mathcal{T}_0(n_0)$ has only one component, but path connectivity is compromised ($b_0 = 1$, $b_1 \gg 1$). In higher dimensions, $\mathcal{T}_0(n_0)$ is contractible, $b_{n>1} = 0$. (**B**) After applying the correction algorithms, the selected complexes for almost all maps acquire correct topological signature in $1D$ and $2D$ (shown in boldface) for $n_0 \geq 7$. The corresponding learning times, $T_{\min}$, are listed in Suppl. Table 4.

A. Original

n_0 **= 2**	n_0 **= 5**	n_0 **= 7**	n_0 **= 12**
29 0	1 31 0 0	1 11 0 0	1 4 1 0
29 0	1 30 0 0	1 16 0 0	1 3 0 0
32 0	1 28 0 0	1 19 0 0	1 3 0 0
31 0	1 31 0 0	1 14 0 0	1 2 0 0
31 0	2 34 0 0	1 20 0 0	1 4 0 0
34 0	1 26 0 0	1 12 0 0	1 2 0 0
28 0	1 41 0 0	1 11 0 0	1 2 0 0
28 0	1 40 0 0	1 15 0 0	**1 1 1 0**
31 0	1 32 0 0	1 17 0 0	1 4 0 0
43 0	1 33 0 0	1 16 4 0	1 4 0 0

B. Corrected

n_0 **= 2**	n_0 **= 5**	n_0 **= 7**	n_0 **= 12**
1 2	**1 1 3 0**	**1 1 1 0**	**1 1 1 1**
**1 1**	1 2 0 0	**1 1 1 0**	**1 1 1 0**
1 4	1 2 0 0	**1 1 0 1**	**1 1 1 0**
1 3	1 4 0 0	**1 1 1 0**	**1 1 1 0**
1 3	1 2 0 0	**1 1 1 0**	1 2 2 1
1 2	1 5 0 0	**1 1 1 0**	1 2 0 1
1 4	1 2 0 0	1 2 1 0	1 2 0 0
1 3	**1 1 0 0**	**1 1 2 0**	**1 1 0 2**
1 3	1 2 0 0	1 3 1 0	**1 1 0 0**
1 3	1 2 0 0	**1 1 5 0**	1 2 0 0

Supplementary Table 4. The learning times, $T_{\min}$ (in minutes) computed for the selected simplicial complexes $\mathcal{T}_0(\theta)$ and $\mathcal{T}_0(n_0)$ are similar to the learning times computed via the full temporal nerve complex $\mathcal{T}$. Thus, the information about the topological structure of the environment emerges from the cell assembly activity as fast as from the entire pool of place cell coactivities. However, note that the complex $\mathcal{T}_0(n_0)$ sometimes fails to produce a finite leaning time; non-convergent cases are marked by ∞.

	1	2	3	4	5	6	7	8	9	10	mean/std
$\mathcal{T}_0$	4.4	2.7	2.3	2.7	2.8	2.7	2.1	3.8	10.7	2.7	3.7/2.5
$\mathcal{T}_0(\theta = .05)$	3.8	2.7	1.9	2.7	3.6	3.8	3.8	3.8	2.5	3.7	3.2/0.7
$\mathcal{T}_0(\theta = .07)$	2.1	3.8	3.8	2.8	2.8	2.3	2.4	3.7	2.5	3.8	3.0/0.7
$\mathcal{T}_0(n_0 = 7)$	1.9	4.6	2.4	2.7	2.8	3.8	∞	3.8	∞	3.8	3.2/0.9
$\mathcal{T}_0(n_0 = 12)$	3.8	2.7	1.9	2.7	∞	∞	∞	3.8	3.8	∞	3.1/0.8

3 SUPPLEMENTARY MOVIES ILLUSTRATING THE FIRST OF THE TEN TESTED MAPS

Suppl. Movie 1. The grey dots represent centers of the place fields, viewed from above, similar to Figure 3B. The centers of the place fields that correspond to the coactive place cells are shown in red. The resulting activity packet moves in the environment following the simulated rat's trajectory.

Suppl. Movie 2. Selection of the cell assemblies by Method I ($\theta = 100$) and assigning readout neurons to the cell assemblies.

Suppl. Movie 3. A side projection view of the activity packet propagating in the cell assembly network, selected via Method I. To emphasize that the coactive place cell combinations comprise a cell assembly complex $\mathcal{T}_0(\theta)$, the corresponding place field centers are schematically connected to the readout neurons.

Suppl. Movie 4. The same system shown in the same projection as the Figure 3B and Suppl. Movie 1.
